# Evaluation of Danazol, Cyclosporine, and Prednisolone as Single Agent or in Combination for Paroxysmal Nocturnal Hemoglobinuria

**DOI:** 10.4274/Tjh.2012.0199

**Published:** 2013-12-05

**Authors:** Kanjaksha Ghosh, Manisha Madkaikar, Maya Gupta, Farah Jijina

**Affiliations:** 1 National Institute of Immunohaematology, 13th Floor, NMS Building, KEM Hospital Campus, Parel, Mumbai, India and Department of Haematology, 10th Floor, NMS Building, KEM Hospital Campus, Parel, Mumbai, India

**Keywords:** cyclosporine, Danazol, Hemoglobinuria, Paroxysmal, Immunosuppression, Prednisolone

## Abstract

**Objective: **The responses of 32 patients with paroxysmal nocturnal hemoglobinuria (PNH) were assessed after the patients were put on various combinations of danazol, prednisolone, and cyclosporine.

**Materials and Methods:** Nineteen males and 13 females aged between 14 and 60 years with confirmed diagnosis of PNH were treated with danazol (4), danazol + cyclosporine (7), cyclosporine (1), and prednisolone + danazol (20). Response to these interventions was assessed regularly. Danazol was added to cyclosporine in patients with aplastic bone marrow after 3 months of cyclocporine use only unless the former therapy was successful. Four patients with aplastic marrow received only danazol because they had renal insufficiency at presentation. Patients were evaluated with regular complete blood count and routine liver and renal function tests.

**Results:** One patient responded to cyclosporine only. Thirteen of 32 patients (40%) had complete response, 12/32 patients (37%) had partial response leading to freedom from red cell transfusion, and 2/32 (7%) had no response. Five patients (16%) died due to thrombosis or hemorrhage within 3 months of therapy before their response to therapy could be assessed. The median period of review of the cases was 4 years and 6 months.

**Conclusion:** Danazol is a useful addition to PNH therapy both in combination with cyclosporine for hypoplastic PNH and with prednisolone for other forms of PNH, and this therapy could be a good alternative where eculizumab and anti-lymphocyte globulin cannot be used for various reasons.

**Conflict of interest:**None declared.

## Abstract (tr)

**Amaç:** Tek başına veya birlikte kullanılan danazol, siklosporin ve prednizolonun tedavideki yeri 32 paroksismal nokturnal hemoglobinürili (PNH) hasta üzerinde değerlendirildi.

**Gereç ve Yöntemler:** Yaşları 14 ile 60 arasında değişen 19 erkek ve 13 kadın PNH tanılı hasta danazol (4), danazol + siklosporin (7), siklosporin (1), ve danazol + prednizolon (20) ile tedavi edildi. Aplastik kemik iliği olup en az 3 aylık siklosporin tedavisine yanıt alınamayan hastalarda tedaviye danazol eklendi. Aplastik kemik iliği olan 4 hastada tanı anında böbrek yetersizliği bulunması nedeniyle sadece danazol kullanıldı. Hastalar düzenli kan sayımı, karaciğer ve böbrek testleri ile değerlendirildi.

**Bulgular:** Bir hastada tek başına siklosporin kullanımına yanıt alındı. Otuz iki hastanın 13’ünde (%40) tam remisyon elde edildi. On iki hastada (%37) kısmi yanıt görüldü ve bu hastalarda eritrosit replasman gereksinimi ortadan kalktı. İki hastada ise (7) tedaviye yanıt alınamadı. Beş hasta (%16) tedavinin ilk 3 ayı içinde yanıt değerlendirmesi yapılamadan tromboz veya kanamadan kaybedildi. Ortanca takip süresi 4,5 yıldı.

**Sonuç: **Danazol hipoplastik PNH’da siklosporine, diğer PNH türlerinde ise prednizolonun yanına eklenebilecek yararlı bir tedavi tamamlayıcı ajan olarak görünmektedir. Özellikle eculizumab ve antilenfosit globulinlere erişimin kısıtlı olduğu yerlerde uygulanabilecek bir tedavi seçeneği oluşturmaktadır.

## INTRODUCTION

Paroxysmal nocturnal hemoglobinuria (PNH) has been variously managed with immunosuppressive therapies such as corticosteroids and cyclosporine [1,2]. Unusual medicines like metronidazole [3] have also been used for this condition. Recently the humanized monoclonal antibody against the C5a component of the complement system (eculizumab) has been very successfully used to manage such cases [4], but such therapy has severe complications apart from the cost, which cannot be borne by average people in developing countries. Hence, there is a need for evaluating common medicines singly or in combination in PNH cases. 

PNH is an uncommon disorder; hence, not many large series on treatment options for such patients are available. Standard management like packed red cell transfusion, iron replacement where indicated, and relevant management for bleeding and thrombotic episodes is applicable to PNH patients along with specific therapy. In this study, 32 well-characterized PNH patients are described as to their outcomes with cyclosporine, steroids, and danazol therapy singly or in various combinations. 

## MATERIALS AND METHODS

**Patients**

Thirty-two patients (19 males and 13 females) aged between 14 and 60 years (median age: 35 years) were recruited for the study over a 4-year period and were followed for at least 1.5 years from the time of diagnosis. The diagnosis of PNH was made on the basis of paroxysmal nocturnal hemoglobinuria, pancytopenia, unusual thrombotic jaundice, anemia, and splenomegaly coupled with low leukocyte alkaline phosphatase score, and the diagnosis was confirmed with positive Ham’s test results and the presence of more than 10% red cells and/or neutrophils from peripheral blood showing negative staining for both CD55 and CD59 monoclonal antibodies. The test was done on a Becton Dickinson FACSCalibur flow cytometer with antibodies supplied by the same company (Becton Dickinson, USA). 

The study was sanctioned by the institutional ethics committee (IEC) of the hospital and informed consent from the patients was taken as is mandatory for sanctioning of studies by the IEC. 

**Investigations**

The patients were tested for complete blood counts, reticulocyte count, bone marrow aspirate for morphology and iron stain, cytogenetic study, and routine biochemistry. Urinary hemosiderin staining was done in addition to Ham’s test and flow cytometry tests to confirm the diagnosis. Other imaging studies were done as per requirements of individual cases. 

**Criteria of Response**

Complete response was defined as an increase in hemoglobin to >110 g/L without red cell support, absolute neutrophil count above 1.5x10^9^/L, platelet count above 100x10^9^/L, and no requirement for blood product support or any infection. Partial response was defined as freedom from red cell support but platelet and neutrophil counts that had not normalized. 

**Therapy**

Supportive management was given to patients in the form of packed red cell transfusion whenever hemoglobin fell below 70 g/L. Bleeding episodes were treated with random donor platelet infusions whenever required. Responses to therapy were assessed every 3 months and patients who did not respond at the end of 3 months were deemed as non-responders to that therapy. 

If the patient had bone marrow aplasia as a part of PNH then the patient was started on cyclosporine at 6 mg/kg daily per os (PO), and in the event of non-response or partial response at the end of 3 months, danazol at 100 mg twice daily PO was added. Oral danazol was titrated up to 400 mg/day if required. Four patients with aplastic marrow who presented with renal insufficiency (serum creatinine of >170 µmol/L) were treated only with danazol. PNH patients who did not have marrow hypoplasia were treated with oral prednisolone at 1 mg/kg PO along with danazol at 100 mg twice daily, which was slowly increased to 400 mg/day if there was no response. After response was achieved, oral corticosteroids were tapered to a level of 7.5-10 mg PO daily over a course of 6-8 weeks. 

If the patient had a prior thrombotic episode and there was no response with either prednisolone and cyclosporine, the patient was warned about the risk of danazol in precipitating thrombosis. If the platelet count was greater than 50x10^9^/L, the patient was started on thromboprophylaxis with low-dose warfarin to maintain an international normalized ratio (INR) of 1.5-2.0 along with danazol at the lowest possible dose. 

The patients had imaging and other studies done as per the requirements of their individual cases. In addition, all patients received a combination of oral iron and folic acid in addition to immunosuppressive and danazol therapy. 

## RESULTS

The clinical presentations of the 32 patients are given in Table 1. Twelve patients presented with marrow hypoplasia and they received either danazol or immunosuppressive therapy, or immunosuppressive therapy and danazol. 

Twenty patients did not have any marrow hypoplasia and they were started with a combination of corticosteroids and danazol orally. The details of these patients are presented in Table 2. 

During the course of follow-up, 5 patients died (16%). One died due to massive gastrointestinal bleeding, 2 due to stroke, 1 due to cerebral venous thrombosis, and 1 due to intraventricular (cardiac) thrombosis. Out of 32 patients, 13 (40%) had complete response and 12 had (38%) partial response, while 2 patients, i.e. 1 receiving only danazol and 1 receiving danazol and prednisolone, did not respond. Hence, 25/32 (78%) patients with PNH had some response to therapy. There were 4/13 complete responses in hypoplastic PNH (33%) cases compared to 9/20 (45%) in PNH cases without marrow hypoplasia. This difference was statistically significant (chi-square test, p<0.01). Twelve out of 32 patients (38%) needed interruption of treatment because of danazol-induced derangement of liver function (10/24) or cyclosporine-induced renal dysfunction (2/8). This interruption was temporary and did not prevent continuation of therapy. Nine patients had evidence of stroke at presentation. 

Four patients with hypoplastic marrow had prior thrombosis, and all of them had low platelet counts and did not respond to cyclosporine therapy alone; hence, danazol was added to this group’s treatment without anticoagulation and none of these patients had developed thrombosis upon follow-up. Five patients in the non-aplastic group had thrombosis at presentation and none of them developed any future thrombotic complications. These patients were on low-dose warfarin with a target INR of 1.5-2.0 as their platelet counts were above 30x109/L.

## DISCUSSION

In the present study, 32 patients with confirmed PNH were treated with various combinations of prednisolone, danazol, and cyclosporine. Cyclosporine was used in those patients who had marrow hypoplasia with PNH. Danazol was added to cyclosporine after 3 months if the cyclosporine was ineffective. One out of 8 patients responded to cyclosporine only and 1/4 had complete response to danazol only, while in 2/7 a complete response was seen when danazol was added to cyclosporine. Hence, in hypoplastic PNH either cyclosporine or danazol alone is less effective (12% and 25% response, respectively) than the combination of cyclosporine and danazol (30% response). In other groups in which danazol and oral corticosteroids were used, there were better complete response rates (9/20, 45%; chi-square test, p<0.05), but death was significantly increased in this group: 4/20 (20%) as compared to 1/12 (8%). All of the deaths were due to thrombohemorrhagic complications. 

There are no randomized trials demonstrating the efficacy of oral steroids in decreasing hemolysis in PNH, but they are still used in the treatment of acute episodes due to their immediate effects. However, continued use of high dosages of corticosteroids is associated with substantial side effects and hence the International PNH Interest Group recommends its use in pulse doses for controlling acute attacks of hemolysis [5]. It appears that steroids inhibit the activation of complements by an alternate pathway to prevent hemolysis [6]. In a prior study, the effect of danazol for PNH efficacy was shown in 4/5 cases of classical PNH refractory to other conventional treatments [7]. This result is similar to our results in 4 patients for whom only danazol was used. How danazol works in PNH is not clearly known, but resistance to osmotic lysis is likely to be one of the mechanisms for its activity [7]. Cyclosporine has been used as a treatment modality for hypoplastic anemia in association with other immunosuppressive drugs like anti-thymocytic globulin [8]. Our rationale for giving cyclosporine to our PNH patients along with danazol seems to be fully justified based on the results showing complete or partial response in 7/7 (100%) patients receiving this combination. However, danazol was important in this group because cyclosporine alone, when used without danazol, was useful only in 1/8 patients. The present study shows that hypoplastic PNH is best treated by a combination of cyclosporine with danazol. We could not use anti-thymocytic globulin or anti-lymphocytic globulin for our patients with severely aplastic marrow because of financial constraints, and due to the same reason we could not use eculizumab, which is even more costly than anti-thymocytic globulin and anti-lymphocytic globulin. 

As a majority of our patients received danazol and 4 of them died due to thrombotic complications while on danazol, it may well be argued whether danazol could have precipitated these thrombotic complications. PNH in its natural course generally has a high rate of venous thrombosis, reported to be as high as 39% [9]. Compared to that, our figure was modest: 4/32 died of thrombotic complication (12%) and 9/32 (28%) had thrombosis at presentation. With such a high background rate of thrombosis, it does not appear that danazol increased the rate significantly. 

Eculizumab is a useful medicine for PNH as it not only reduces hemolysis but also reduces the frequency of thrombosis, proving conclusively the role of activated complement components in the pathogenesis of thrombosis in this condition [4]. However, we were not able to use it because of its cost; this is also the case with recombinant erythropoietin, which has been used by some authors in combination with corticosteroids [6]. Eculizumab has its own serious side effects in the form of intense hemolysis if the medicine is stopped suddenly, or bacterial meningitis due to blockade of complement component C5a [4,5]. In the present study, danazol, which was used in 31/32 patients alone or in combination with cyclosporine or prednisolone, led to 13 complete remissions and 12 partial remissions (82% response) with freedom from red cell infusion. Hence, danazol appears to be a useful medicine for the treatment of PNH in developing countries where financial constraints preclude the use of eculizumab. 

## CONFLICT OF INTEREST STATEMENT

The authors of this paper have no conflicts of interest, including specific financial interests, relationships, and/ or affiliations relevant to the subject matter or materials included.

## Figures and Tables

**Table 1:**
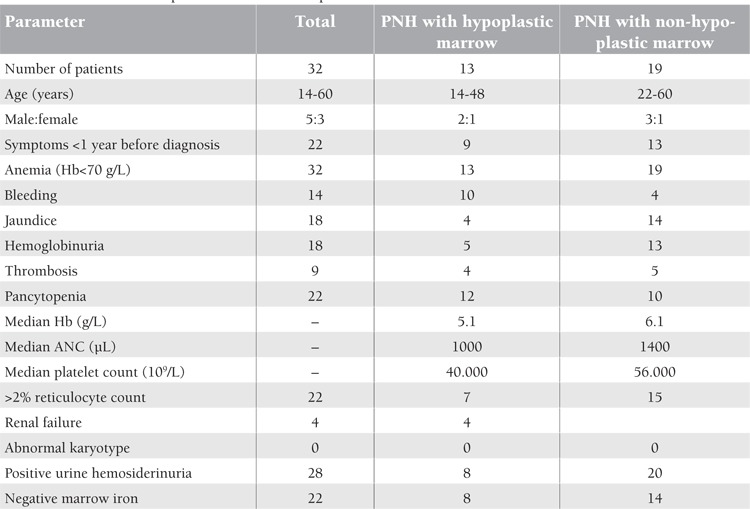
Clinical feature at presentation of 32 PNH patients

**Table 2:**
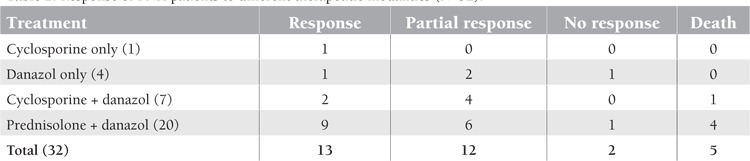
Response of PNH patients to different therapeutic modalities (N=32)
